# From Traditional Inspection to Quantitative Imaging: Tongue and Facial Color Features for Automated Machine Learning-Driven Depression and Schizophrenia Classification

**DOI:** 10.3390/bs16081442

**Published:** 2026-08-20

**Authors:** Limin Gao, Mengmeng Zhang, Yuanhao Li, Lijuan Wang, Peng Qian, Jie Tong, Haojie Fu, Xirong Sun, Fufeng Li

**Affiliations:** 1 Clinical Research Center for Mental Disorders, Shanghai Pudong New Area Mental Health Center, Tongji University, Shanghai 200124, China; 2 Department of General Internal Medicine and Psychosomatics, University Hospital Heidelberg, 69120 Heidelberg, Germany; 3 Shanghai University of Traditional Chinese Medicine, Shanghai 201203, China; 4 Shanghai Research Institute for Intelligent Autonomous Systems, Tongji University, Shanghai 201210, China

**Keywords:** depression, schizophrenia, non-invasive assessment, tongue image, traditional Chinese medicine, automated machine learning

## Abstract

Objective and non-invasive markers for psychiatric assessment remain limited. This study evaluated whether standardized tongue and facial color features provide measurable information relevant to depression and schizophrenia classification. Tongue and facial images were collected from 749 participants, including healthy controls (n = 84), patients with depression (n = 246), and patients with schizophrenia (n = 419). Color characteristics were quantified in predefined tongue and facial regions using the LAB color space. Group differences were examined statistically, and machine-learning models were evaluated across five repeated stratified splits. Most LAB-derived features differed significantly across groups, with luminance-related measures showing the largest effect sizes and more consistent shifts in schizophrenia than in depression. In multiclass classification, LAB plus demographic variables achieved strong performance (accuracy = 0.782 ± 0.035, macro-F1 = 0.697 ± 0.038, AUC = 0.912 ± 0.027), similar to LAB plus demographic and traditional variables (AUC = 0.912 ± 0.032). LAB-only models showed comparable AUC to demographic-only models but lower macro-F1. In pairwise analyses, discrimination was strongest for healthy control versus schizophrenia (AUC = 0.952 ± 0.030) and depression versus schizophrenia (AUC = 0.926 ± 0.019), and lower for healthy control versus depression (AUC = 0.817 ± 0.054). These findings suggest that LAB-derived tongue and facial color features may provide complementary group-level information, particularly when combined with demographic variables, but should not be interpreted as standalone diagnostic biomarkers.

## 1. Introduction

Depression and schizophrenia are among the most prevalent and disabling psychiatric disorders worldwide (Santomauro et al., 2021). Despite their clinical importance, the diagnosis of these disorders largely relies on symptom-based clinical interviews (Aboraya et al., 2006; Nordgaard et al., 2013). Objective biomarkers that are reliable and readily applicable in routine psychiatric practice remain limited (Abi-Dargham et al., 2023; Kas et al., 2025). Although neuroimaging and molecular approaches have advanced psychiatric research, their routine clinical use is constrained by cost, complexity, and accessibility (C. H. Fu & Costafreda, 2013; McCutcheon et al., 2026). This gap has motivated growing interest in non-invasive and easily obtainable indicators that may complement existing diagnostic approaches. External physical features may provide additional cues relevant to mental health assessment (Hostalet et al., 2024; Xu et al., 2022). Such perspectives resonate with holistic medical traditions that emphasize the relationship between external appearance and internal regulation (Dong, 2013; Matos et al., 2021b). Within this context, traditional medical interpretations of mental disorders offer a distinct perspective on the potential relevance of external physical features.

In traditional Chinese medicine (TCM), emotional activities (qingzhi) are considered closely related to the functional states of the visceral systems (zangfu), and prolonged emotional dysregulation is described as a potential contributor to systemic imbalance (Ye et al., 2019). Within this theoretical framework, disturbances of qi and blood are often used to describe altered physiological regulation that may manifest as observable external signs. Tongue inspection is a core component of TCM observation and has standardized terminology for describing tongue body and coating features (Jung et al., 2012). In recent years, tongue diagnosis has also been increasingly operationalized using digital imaging, where quantitative descriptors such as tongue color, coating color, coating thickness, and moisture are extracted under controlled acquisition and color-calibration procedures (Q. Liu et al., 2023; Xie et al., 2021). This combination of traditional observational concepts and modern image-based quantification motivates the investigation of whether tongue (and related facial) appearance features contain measurable signals associated with psychiatric conditions, while acknowledging that such associations require empirical validation (Lo et al., 2012; Zhang et al., 2021).

Despite its long history, traditional tongue and facial inspection relies heavily on qualitative judgment and clinical experience (Segawa et al., 2023). This subjectivity limits reproducibility and poses challenges for systematic scientific investigation (Y. Liu et al., 2025). Advances in digital imaging enable objective characterization of tongue and facial appearance (Xie et al., 2021). In particular, color information constitutes a central component of tongue and facial assessment. The adoption of standardized color spaces allows color information to be represented as continuous numerical variables (Kawanabe et al., 2016; Xie et al., 2021). However, the application of such quantitative tongue and facial features to psychiatric disorders remains limited. This gap warrants further evaluation of quantitative tongue and facial features in psychiatric assessment (Han et al., 2024).

Depression and schizophrenia are increasingly recognized as disorders involving widespread dysregulation across emotional, autonomic, and physiological systems (Santamaría-García et al., 2025; Wang et al., 2025). Similar ideas have been articulated in holistic medical frameworks that emphasize external signs of internal imbalance (Lo et al., 2012; Matos et al., 2021a). The tongue and face are highly vascularized and richly innervated, making them sensitive to changes in systemic regulation (Mu & Sanders, 2010). Several biological pathways may provide a plausible, although still exploratory, link between psychiatric disorders and tongue/facial color characteristics (Alvares et al., 2016). Dysautonomia may affect peripheral vascular tone and microcirculatory perfusion, which could influence luminance and red–green color components in facial and lip regions and may also be relevant to tongue appearance through mucosal perfusion (Ly et al., 2020). Chronic inflammation and oxidative stress, both reported in severe psychiatric conditions, may also be associated with systemic metabolic and vascular changes that affect mucosal and skin appearance. In addition, altered salivary secretion, oral condition, hydration, medication exposure, and self-care may influence tongue coating, gloss-related features, and brightness (Cockburn et al., 2017; Wolff et al., 2017). These pathways do not imply disease-specific visual biomarkers, but they provide a biologically plausible rationale for examining whether standardized LAB-derived tongue and facial features show group-level differences across clinical reference groups. Color-related characteristics, in particular, represent a salient dimension of such external manifestations (Tania et al., 2019). Depression and schizophrenia differ not only in core psychopathological symptoms, but also in typical patterns of psychomotor activity, autonomic regulation, sleep disturbance, medication exposure, self-care, and broader physiological burden (Stogios et al., 2021). These factors may influence facial complexion, lip color, tongue coating, moisture-related appearance, and gloss through changes in peripheral circulation, hydration, oral condition, and behavioral state (Cockburn et al., 2017; Ly et al., 2020). Therefore, it is reasonable to explore whether quantitative tongue and facial color features show diagnosis-related group-level patterns across these disorders, while recognizing that such features should not be interpreted as disorder-specific diagnostic biomarkers. To reduce investigator degrees of freedom in manual model selection and hyperparameter tuning, we adopted an automated machine learning (AutoML) workflow to systematically compare multiple candidate classification algorithms under a unified and reproducible pipeline. AutoML has been advocated as a practical approach for research settings where predictive modeling aims to (i) automate hyperparameter optimization, (ii) identify influential features via model interpretability methods, and (iii) facilitate transparent model selection procedures that support theory building and empirical validation (H. Fu & Zhao, 2025). In the present study, the AutoML procedure was applied to each predefined feature set using identical data splitting and preprocessing steps. Candidate models were trained within the training data only, and the best-performing model was selected based on performance on the internal validation procedure using the primary metric defined a priori. The selected model was evaluated on the held-out test set for each repeated split, and performance was summarized across the five seeds. Random seeds were fixed across data splitting and model training to ensure reproducibility (H. Fu & Zhao, 2025).

Accordingly, we conducted a three-level evaluation of tongue and facial color features in depression and schizophrenia. At the statistical level, tongue and facial color characteristics were quantified using standardized CIE LAB features and examined for group differences and effect sizes across diagnostic categories (Ly et al., 2020). At the machine-learning level, predictive models were trained to test whether these quantitative features provide discriminative information for diagnostic classification under both multiclass and pairwise binary settings. At the interpretability level, feature contribution patterns were analyzed to identify which color features consistently drive model decisions and to compare shared versus diagnosis-specific signatures across modeling configurations. By integrating statistical inference, predictive modeling, and interpretability analyses, this study provides an exploratory framework for quantifying tongue and facial phenotypes in psychiatry and for clarifying their potential role as complementary, non-invasive indicators of system-level dysregulation.

## 2. Materials and Methods

### 2.1. Study Design and Participants

A cross-sectional study design was employed in the present investigation. Participants were recruited from a psychiatric outpatient service in Pudong New District, Shanghai. Psychiatric group labels were based on routine outpatient diagnoses assigned by licensed psychiatrists and were used as clinical reference labels in the machine-learning analyses. Because the study was based on real-world clinical practice, the diagnostic labels reflect routine psychiatric evaluation rather than diagnoses established through a structured research interview. Accordingly, these labels should be interpreted as clinician-assigned clinical reference labels rather than definitive research diagnostic ground truth categories. The study followed a sequential workflow from image acquisition to feature extraction and predictive analysis. After enrollment, participants underwent standardized facial and tongue image acquisition during their outpatient visit. The collected data consisted primarily of tongue and facial images, which served as the raw input for subsequent analysis.

### 2.2. Image Acquisition and Quality Control

Standardized procedures were implemented for facial and tongue image acquisition to ensure data quality and consistency. All images were collected in the outpatient clinic under controlled and consistent environmental conditions using a mobile imaging device based on the portable intelligent mirror “Yunzhongyi” (version 1.0; Beijing Xima Medical Technology, Beijing, China) image acquisition framework (Guo et al., 2021). In previous applications of this system, facial and tongue images and questionnaire information were collected online through the portable intelligent mirror platform, and the resulting data were exported for subsequent analysis. To reduce the influence of ambient-light variation, images were acquired under a standardized setting, with the auxiliary light source of the portable intelligent mirror turned on during acquisition. Facial and tongue images were acquired in a fixed order for all participants. During acquisition, participants were instructed to maintain a stable seated posture and avoid unnecessary movement; for tongue imaging, they were asked to fully extend the tongue to ensure adequate visualization. Only images meeting predefined quality requirements were included in subsequent analyses. Images were excluded if participant information was incomplete, if the facial or tongue image was unclear, or if reliable image detection could not be completed because of factors such as inappropriate posture, excessive hair covering the face, incomplete tongue extension, blurred, partially occluded, improperly framed, or failed to capture the required facial or tongue regions. Images that did not meet these criteria were excluded before feature extraction.

### 2.3. Image Processing, Region Definition, and LAB Feature Extraction

After acquisition, images underwent standardized preprocessing, color correction, noise filtering, and image quality optimization prior to region definition and feature extraction. The image-processing workflow was based on the established “Yunzhongyi” facial and tongue image analysis framework, in which raw images are first preprocessed and color-corrected, followed by facial/tongue region segmentation, tongue-body and tongue-coating separation, and digital feature extraction. Color characteristics were quantified in the LAB color space, in which L represents luminance and a and b represent the red-green and yellow-blue color axes, respectively. Compared with device-dependent color models, LAB provides a more perceptually uniform and device-independent representation of color and is therefore suitable for standardized quantitative analysis. Region-based definitions were applied to both facial and tongue images. Facial images were partitioned into regions corresponding to complexion, lip color, and facial gloss, whereas tongue images were divided into tongue body and coating regions. Consistent with previous applications of this system, facial features included complexion, lip color, and facial gloss, while tongue features included tongue color, coating color, and coating thickness. LAB parameters were used to quantify complexion, lip color, tongue color, and coating color; facial gloss was categorized as glossy, slightly glossy, or non-glossy, and coating thickness was categorized as thick or thin. LAB features were then extracted separately from each predefined region to ensure systematic and comparable quantification across participants (Ly et al., 2020).

### 2.4. Statistical Analysis of LAB Features

One-way analysis of variance (ANOVA) was used to test overall group effects for each LAB feature. Effect sizes were quantified using eta-squared (η^2^) to estimate the proportion of variance explained by group membership. Effect sizes were reported to complement significance testing. An adjusted *p*-value threshold of 0.05 was used to determine statistical significance. Statistical findings were subsequently used to contextualize modeling results.

### 2.5. Machine Learning-Based Prediction

Machine learning models were constructed to evaluate the predictive value of LAB-based features for classification against clinician-assigned clinical reference labels. Classification analyses included a three-class setting as well as pairwise binary comparisons. Multiple feature sets were evaluated, including LAB features, demographic variables, traditional Chinese medicine (TCM)-related image and questionnaire features, and their combinations. Demographic variables included age, sex, height, and body weight. TCM-related features were derived from the standardized facial, tongue, and questionnaire information collected through the image acquisition platform. Based on the established feature framework, these variables included facial gloss and coating thickness as categorical image-derived variables, together with questionnaire-based TCM assessment variables recorded during routine data collection. LAB parameters were treated as continuous variables, whereas facial gloss and coating thickness were treated as categorical variables. The complete list of input features is provided in Supplementary Table S1. Combined models incorporated LAB features with demographic variables, TCM features, or both. An automated machine learning (AutoML) framework was used to train and compare classification models. All preprocessing steps were incorporated into the automated modeling workflow. We formulated the prediction task at two complementary levels. First, a multiclass classification setting (depression vs. schizophrenia vs. healthy control) was used to evaluate the overall discriminability of quantitatively derived tongue and facial features with respect to the clinician-assigned clinical reference groups, in a scenario that better reflects real-world differential assessment. Second, we conducted pairwise binary classifications (depression vs. healthy control; schizophrenia vs. healthy control; depression vs. schizophrenia) to address clinically specific decision boundaries and to avoid potential masking effects of macro-averaged multiclass metrics, whereby strong performance on one contrast may obscure difficulties on another. The binary analyses also facilitate imbalance-aware evaluation (e.g., AUPRC) and clearer interpretation of error patterns for each contrast.

Multiclass and pairwise binary prediction models were developed using AutoGluon 1.5.0 with the best_quality preset. Candidate base learners included gradient boosting machines (LightGBM, CatBoost, XGBoost), ensemble tree methods, and neural networks. The framework automatically performed hyperparameter search, model selection, and ensemble construction via bagging and multi-layer stacked generalization, yielding a final weighted ensemble for each feature set and classification task (Erickson et al., 2020).

### 2.6. Evaluation Metrics and Validation Strategy

The dataset comprised 749 samples, which were partitioned into training, validation, and held-out test sets using stratified random sampling to preserve class proportions across all splits. To evaluate the stability of model performance and reduce dependence on a single random split, all predictive analyses were repeated across five random seeds: 42, 123, 777, 2024, and 3407. For each seed, 15% of the data were held out as the test set, and the remaining samples were divided into training (70%) and validation (15%) sets. To prevent data leakage, the independent test set was withheld throughout all stages of model development; only the training and validation sets were made available to AutoGluon for fitting and hyperparameter optimization. To mitigate the effect of class imbalance (healthy controls (HC):depression:schizophrenia ≈ 1:2.9:5.0), inverse-frequency sample weights were applied during training. Model performance was evaluated using complementary metrics appropriate for multi-class and imbalanced classification, including accuracy, balanced accuracy, macro-averaged precision, recall, and F1-score, as well as the area under the receiver operating characteristic curve (AUC) and the area under the precision–recall curve (AUPRC) (Brodersen et al., 2010; Davis & Goadrich, 2006; Fawcett, 2006; Hand & Till, 2001; Saito & Rehmsmeier, 2015). Multiclass AUC was computed using a one-vs-rest weighted-average scheme. For each model, performance metrics were summarized as the mean and standard deviation across the five seeds. In ROC visualizations, shaded regions represent ±1 standard deviation across the five repeated runs. Feature importance was quantified via permutation importance computed on the held-out test set using the full ensemble predictor.

## 3. Results

### 3.1. Sample Characteristics

A total of 749 participants were included in the analysis, comprising 84 healthy controls, 246 patients with depression, and 419 patients with schizophrenia (Table 1). Participants with depression had the lowest mean age (28.94 ± 18.59 years), whereas those with schizophrenia had the highest mean age (53.92 ± 11.82 years). Average height was similar across groups, ranging from 164.39 ± 8.04 cm to 166.30 ± 8.30 cm. The schizophrenia group showed the highest mean body weight (70.72 ± 13.58 kg), followed by the HC group (64.39 ± 16.45 kg) and the depression group (60.37 ± 13.04 kg). The proportion of males was 67.9% (57/84) in the HC group, 71.5% (176/246) in the depression group, and 47.0% (197/419) in the schizophrenia group.

### 3.2. Group Differences in LAB Tongue Features and Facial Features

**Figure 1:**
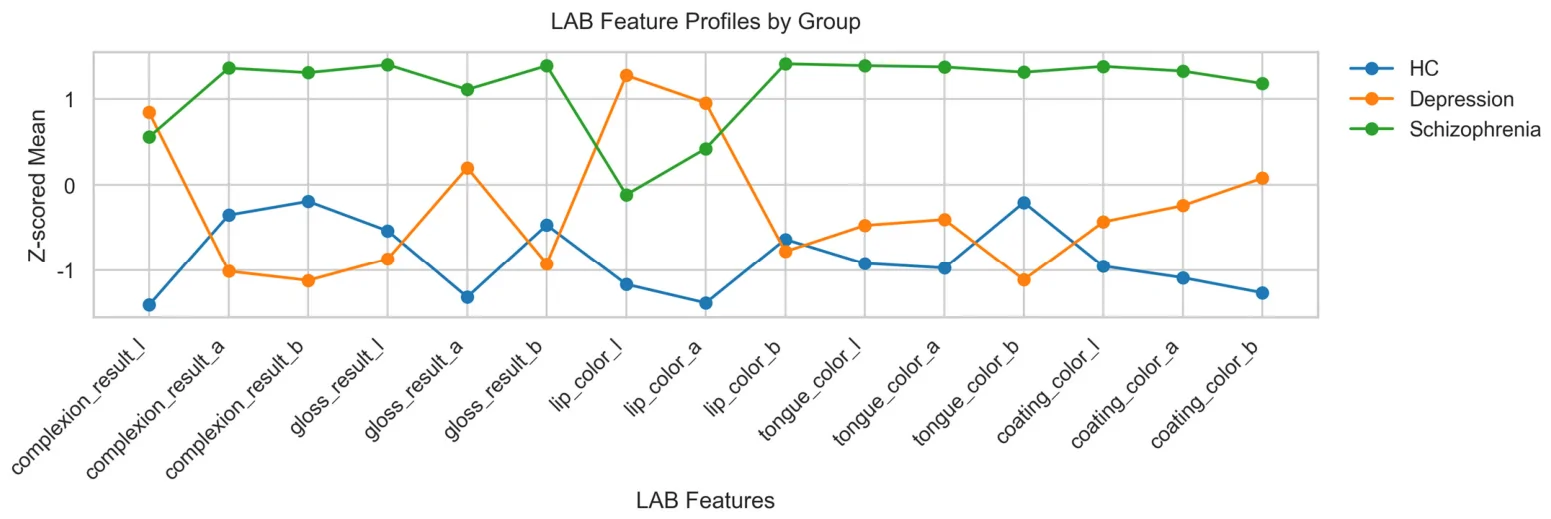
Z-score profiles of LAB tongue and facial color features by diagnostic group. Legend: Group-wise mean Z-scores are shown for each LAB feature. Positive values indicate higher feature levels relative to the overall sample mean, and negative values indicate lower levels.

**Figure 2:**
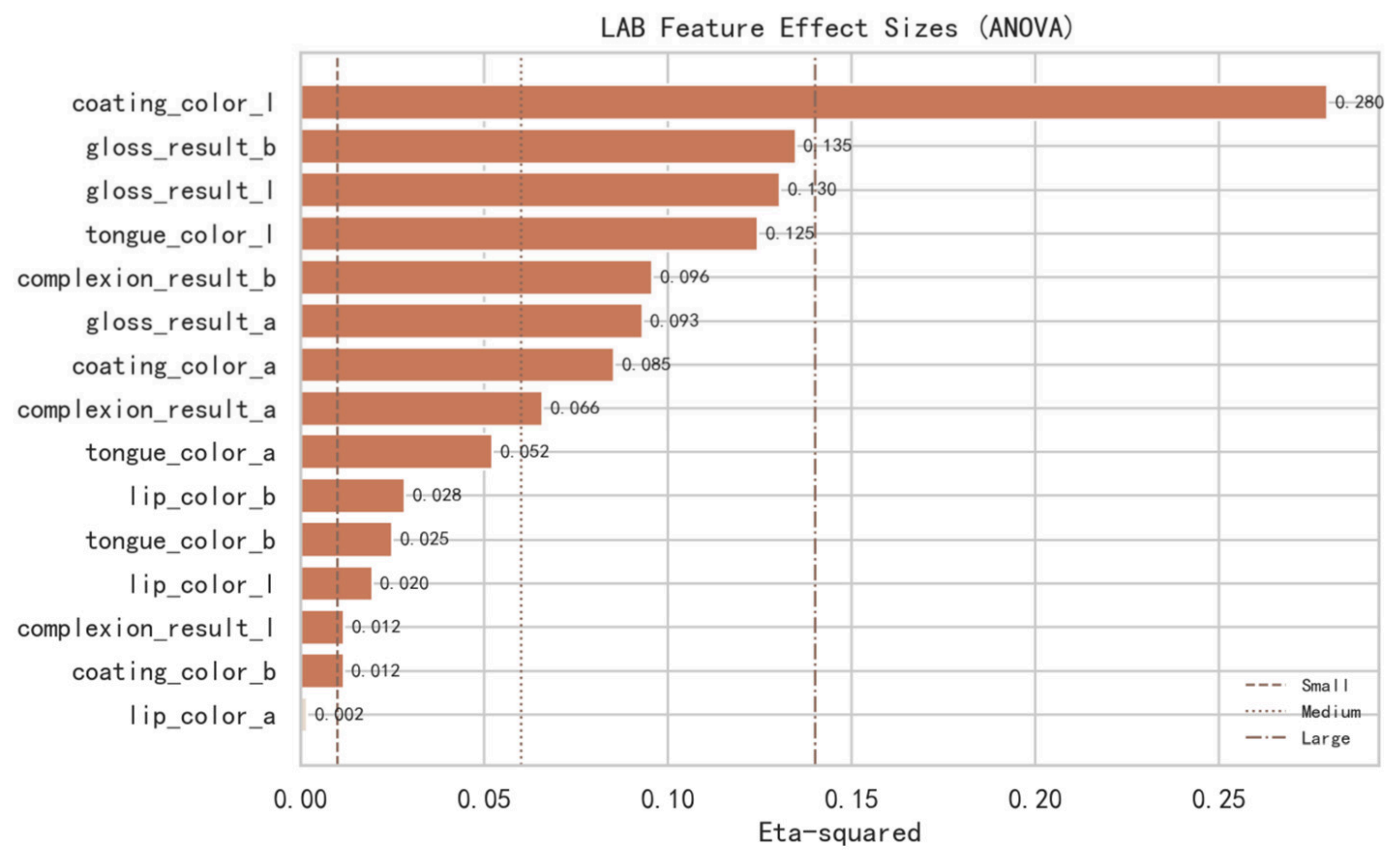
Effect sizes (η^2^) of LAB features for group differences. Legend: Bars represent eta-squared (η^2^) values from one-way ANOVA for each LAB feature. Dashed vertical lines indicate conventional thresholds for small, medium, and large effects.

**Figure 3:**
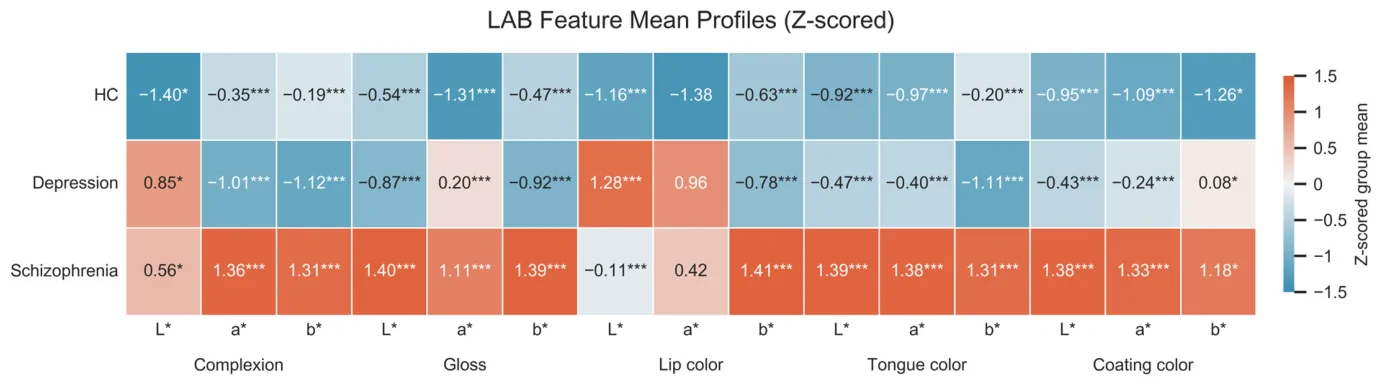
Heatmap of mean Z-scored LAB features across diagnostic groups. Legend: Colors indicate mean Z-scores for each feature within each diagnostic group. Asterisks denote features with statistically significant overall group effects (* *p* < 0.05, *** *p* < 0.001).

Distinct group-level differences were observed across LAB tongue and facial color features. To facilitate comparison across features, group means were standardized using Z-scores. Across most LAB features, the schizophrenia group exhibited positive Z-scores, indicating higher values relative to the overall sample mean (Figure 1). One-way ANOVA revealed significant group effects for the majority of LAB tongue and facial color features (Figure 2). Among all features, coating L showed the largest effect size (η^2^ = 0.28), followed by the gloss-related features b (η^2^ = 0.135) and l (η^2^ = 0.130), and tongue-color L (η^2^ = 0.125). Features related to luminance (L*) consistently showed larger effect sizes than chromatic components (a* and b*). Figure 3 summarizes mean Z-scores of all LAB features for each diagnostic group. Consistent with the profile plot, the schizophrenia group showed uniformly higher Z-scores across most tongue, coating, and gloss features. Mean Z-scores in the depression group were generally lower than those in the schizophrenia group but higher than those in the HC group.

### 3.3. Multiclass Classification Performance Across Feature Sets

**Figure 4:**
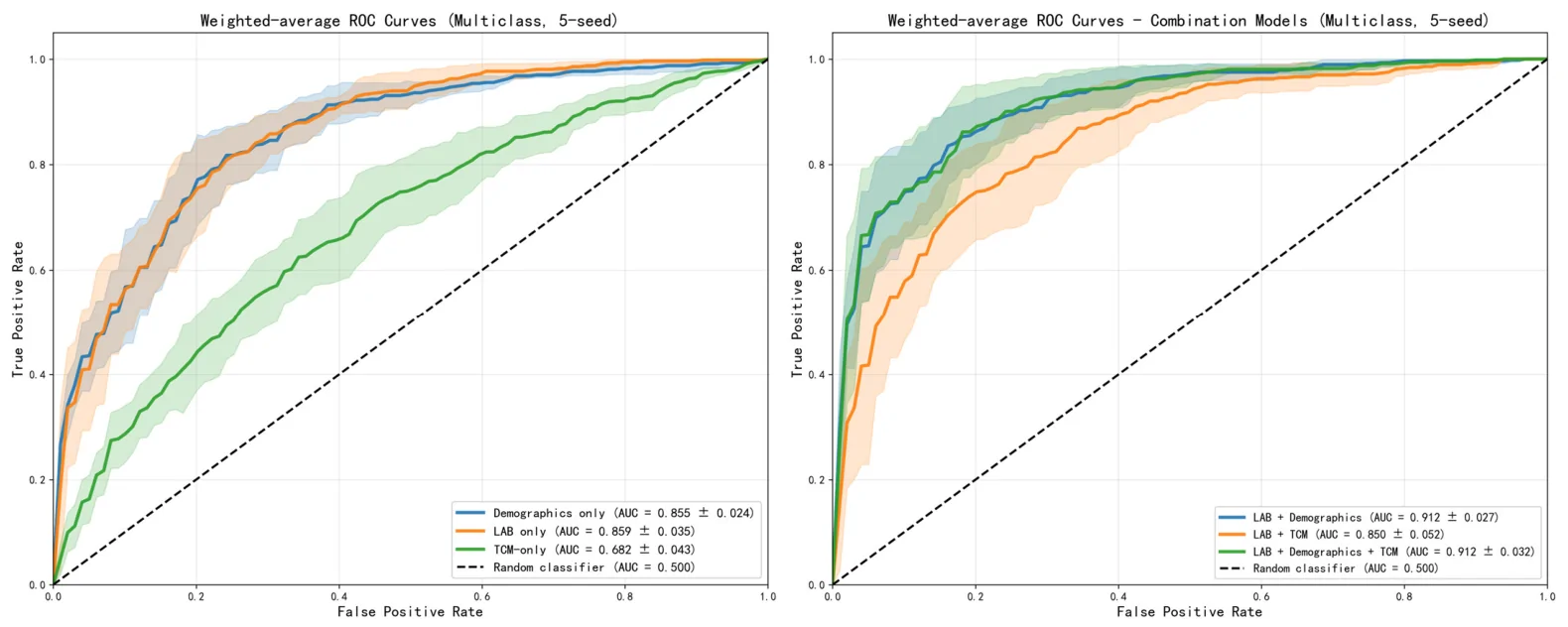
Weighted-average ROC curves for multiclass classification across feature sets. ROC curves are shown for single-feature models and combined-feature models across five repeated stratified splits. Solid lines represent the mean weighted-average ROC curve across the five random seeds, and shaded regions represent ±1 standard deviation. The dashed diagonal line indicates random classification performance. AUC values are reported as mean ± standard deviation across the five seeds.

Multiclass analyses were first conducted to assess overall separability across the three diagnostic groups, followed by pairwise analyses of contrast-specific discriminability (Table 2, Figure 4). Across five repeated stratified splits, the demographic-only model showed relatively strong multiclass performance (accuracy = 0.754 ± 0.036, balanced accuracy = 0.631 ± 0.046, macro-F1 = 0.639 ± 0.046, AUC = 0.855 ± 0.024, AUPRC = 0.805 ± 0.028). The LAB-only model achieved comparable AUC but lower classification accuracy and macro-F1 (accuracy = 0.704 ± 0.040, balanced accuracy = 0.554 ± 0.052, macro-F1 = 0.552 ± 0.051, AUC = 0.859 ± 0.035, AUPRC = 0.761 ± 0.053), with only a small AUC difference relative to the demographic-only model (ΔAUC = +0.004). The TCM-only model showed the weakest performance among single-feature models (accuracy = 0.538 ± 0.043, balanced accuracy = 0.467 ± 0.055, macro-F1 = 0.456 ± 0.049, AUC = 0.682 ± 0.043, AUPRC = 0.604 ± 0.037).

When feature sets were combined, LAB + Demographics achieved the highest AUC and AUPRC among the tested configurations (AUC = 0.912 ± 0.027, AUPRC = 0.858 ± 0.016), with improved balanced accuracy and macro-F1 compared with either LAB-only or demographic-only models. The LAB + Demographics + TCM model showed similar performance (accuracy = 0.784 ± 0.051, balanced accuracy = 0.705 ± 0.044, macro-F1 = 0.699 ± 0.045, AUC = 0.912 ± 0.032, AUPRC = 0.858 ± 0.027). By contrast, adding TCM features to LAB features without demographic variables did not improve AUC relative to the LAB-only model (LAB + TCM: AUC = 0.850 ± 0.052; ΔAUC vs. Demo = −0.005). These results indicate that LAB-derived features provided additional predictive information when combined with demographic variables, but they also highlight the substantial contribution of demographic structure to multiclass classification performance.

### 3.4. Performance in Pairwise Diagnostic Comparisons

**Figure 5:**
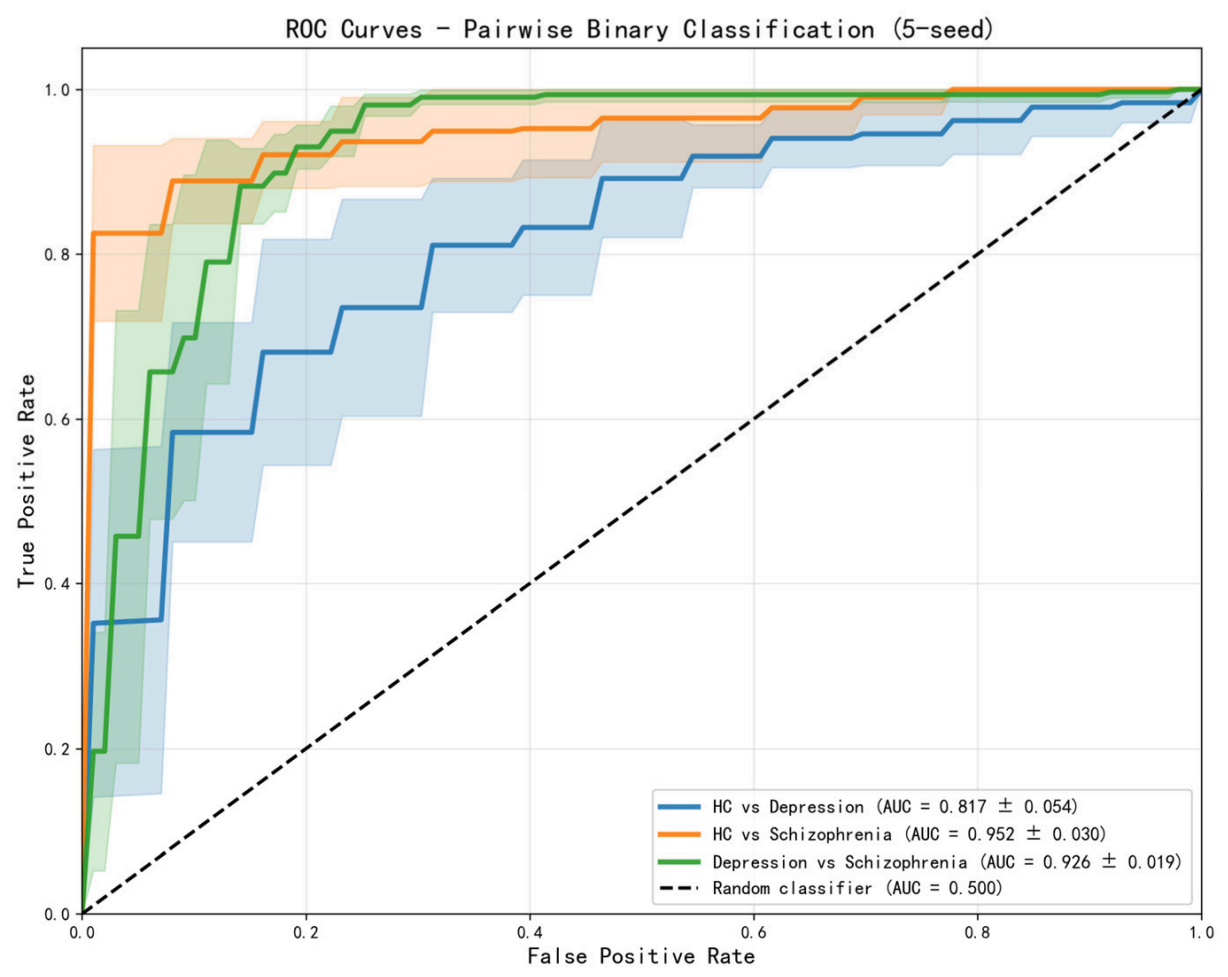
ROC curves for pairwise binary classification across diagnostic contrasts. Solid lines represent mean ROC curves across five repeated stratified splits, and shaded regions indicate ±1 standard deviation across the five random seeds. The dashed diagonal line indicates random classification performance. AUC values are reported as mean ± standard deviation across the five seeds.

Pairwise binary classifications were performed to quantify discriminability for clinically relevant contrasts and to complement the multiclass findings (Table 3, Figure 5). All pairwise binary classification models used the complete LAB + Demographics + TCM feature set. Across five repeated stratified splits, discrimination was lowest for HC versus depression, with an accuracy of 0.752 ± 0.059, balanced accuracy of 0.743 ± 0.030, F1 score of 0.815 ± 0.059, AUC of 0.817 ± 0.054, and AUPRC of 0.925 ± 0.033. For HC versus schizophrenia, performance was higher, with an accuracy of 0.892 ± 0.046, balanced accuracy of 0.898 ± 0.023, F1 score of 0.931 ± 0.032, AUC of 0.952 ± 0.030, and AUPRC of 0.990 ± 0.006. For depression versus schizophrenia, the model also achieved high discriminative performance, with an accuracy of 0.874 ± 0.026, balanced accuracy of 0.870 ± 0.026, F1 score of 0.898 ± 0.022, AUC of 0.926 ± 0.019, and AUPRC of 0.935 ± 0.034. Overall, pairwise classification performance was strongest for contrasts involving schizophrenia and comparatively lower for HC versus depression, indicating that depression-related separability remained more limited than schizophrenia-related separability under the repeated-split evaluation. Additional normalized confusion-matrix results, class-specific precision/recall/F1 scores, and top-ranked permutation-importance variables for the multiclass LAB + Demographics + TCM model are provided in Supplementary Tables S2–S4.

## 4. Discussion

The central finding of this study is that tongue and facial color features exhibit differential relevance for psychiatric classification, with markedly stronger signals for schizophrenia than for depression. Across both group-level analyses and predictive modeling, schizophrenia consistently demonstrated more pronounced and coherent feature patterns than depression. Depression showed comparatively lower separability from HC than schizophrenia-related contrasts across the repeated-split analyses. LAB-based color features contributed meaningful information to classification models, particularly when combined with demographic variables. By contrast, TCM-related features showed more limited standalone predictive value under the current encoding scheme. Taken together, the findings suggest that tongue and facial color features may capture group-level appearance differences across clinician-assigned clinical reference groups, especially for schizophrenia-related contrasts, while requiring cautious interpretation because demographic variables also contributed substantially to model performance.

The present findings suggest that clinician-assigned clinical reference groups differ in their degree of external phenotypic visibility, as reflected by LAB-based tongue and facial features. From the perspective of traditional inspection-based frameworks, external features are understood as reflections of internal regulatory imbalance rather than direct representations of symptoms (Matos et al., 2021a; Qi et al., 2016). The clearer and more coherent LAB patterns associated with schizophrenia may therefore reflect a relatively stable and shared form of internal dysregulation that is externally visible (Cowan et al., 2022). This pattern implies that depressive experiences may be less reliably expressed through tongue and facial appearance alone (Cai et al., 2020; Cowan et al., 2022). Across the pairwise tasks, discriminability differed markedly by contrast. In particular, the HC vs. depression comparison showed lower performance than contrasts involving schizophrenia, suggesting that the visual phenotype captured by the current LAB feature set may be subtle and/or more heterogeneous in depression (Saito & Rehmsmeier, 2015). This pattern is consistent with the view that depression is a broad and clinically heterogeneous construct, which can reduce the presence of a single consistent external signature in cross-sectional imaging data (Buch & Liston, 2021; Nemesure et al., 2024; Simmonds-Buckley et al., 2021).

Rather than mapping one-to-one onto symptom-based diagnostic labels, the present results suggest that LAB-based tongue and facial descriptors may capture broader appearance dimensions that vary with general physiological or behavioral state (Cuthbert, 2014, 2020). This interpretation is supported by the effect-size profile, where coating luminance and gloss-related indices showed the strongest between-group separation, pointing to brightness- and reflectance-related properties as the most salient visual signals in this dataset. Importantly, limited alignment with any single diagnostic category should not be interpreted as a failure of the features per se; instead, it may reflect the complex, overlapping nature of psychiatric phenotypes and the constraints of cross-sectional, image-only measurement (Cuthbert, 2014; Ruggero et al., 2019).

From a traditional inspection perspective, external signs are typically interpreted as reflecting broader constitutional and state-like patterns rather than single symptom clusters. In this context, the comparatively lower separation observed for HC vs. depression may be compatible with a more heterogeneous and state-dependent presentation in depression, whereas coating luminance and gloss-related features appear to provide more stable signals across groups in the current dataset (Kambeitz et al., 2017). However, establishing any mechanistic interpretation requires additional multimodal evidence beyond imaging (Kambeitz et al., 2017; J. Liu et al., 2025).

Finally, the consistent performance gains observed when demographic variables were combined with LAB features indicate that contextual information matters for classification. Across five repeated stratified splits, the demographic-only model already showed relatively strong performance (AUC = 0.855 ± 0.024), while the LAB-only model achieved a comparable AUC (0.859 ± 0.035) but lower balanced accuracy and macro-F1. The LAB + Demographics model improved performance further (AUC = 0.912 ± 0.027), suggesting that LAB-derived features may add predictive information when integrated with demographic variables. At the same time, a parsimonious explanation is that demographic factors capture baseline differences and potential confounding structure in the sample, such as the marked between-group differences in age and sex, thereby improving model calibration and separability when integrated with visual features (Fernandes et al., 2020; J. Liu et al., 2025). Accordingly, demographic confounding remains an important limitation and should be considered when interpreting the findings. The observed improvement in combined models should therefore be interpreted as exploratory evidence of complementary information rather than as proof of demographic-independent diagnostic signal. Future work should therefore adopt stratified analyses and confound-aware modeling strategies, including matching, covariate adjustment, and external validation, to clarify under what conditions LAB-derived phenotypes provide robust value beyond demographic information (Dai et al., 2025; J. Liu et al., 2025). In this framing, image-based LAB features are best understood as complementary signals within a broader assessment framework rather than as standalone diagnostic determinants (Conway et al., 2023; Cuthbert, 2014).

The present findings highlight several boundary conditions that also define the main limitations of automated tongue and facial phenotyping in psychiatry. First, LAB-based tongue and facial features do not map directly onto specific psychiatric symptoms or diagnostic categories. Accordingly, the primary insights derived from LAB features are expressed at the group or structural level rather than at the level of individual diagnosis. Second, the diagnostic groups were defined using clinician-assigned routine clinical diagnoses rather than structured research interviews such as SCID-5 or MINI. Although this approach reflects real-world outpatient practice, it may introduce label uncertainty and potential misclassification in the machine-learning setting. Therefore, the present models should be interpreted as exploratory classifications based on clinical reference labels rather than as validation of definitive diagnostic categories. Third, the marked age and sex differences across diagnostic groups represent a major source of potential confounding and may have contributed to both the observed LAB feature differences and classification performance. Although adding LAB features to demographic variables increased the mean AUC, this does not establish a demographic-independent diagnostic signal. Future studies should use age- and sex-balanced cohorts and confound-aware analytical strategies to evaluate the independent contribution of LAB-derived features. Another limitation arises from the use of static images to characterize conditions that may fluctuate over time. In addition, medication exposure was not systematically modeled in the present analysis. This is particularly relevant for the schizophrenia group, as antipsychotic medication and related side effects may influence salivation, oral condition, facial expression, self-care, body weight, and other factors that could affect tongue coating, facial gloss, and color-related image features. Therefore, medication-related confounding should be considered when interpreting the observed group differences and classification performance. These boundary conditions suggest that future research should emphasize conceptual alignment over maximizing classification performance. Future work may benefit from integrating LAB-based phenotyping with symptom dimensions, contextual variables, and longitudinal observations. Taken together, these considerations position automated tongue and facial phenotyping as a context-dependent, system-level complement to existing psychiatric assessment frameworks. Its value lies in delineating patterns of systemic variation rather than replacing established diagnostic practices. In addition, ethnicity, detailed acquisition metadata such as camera distance and image resolution, and the exact number of excluded images by exclusion reason were not fully retained, which may limit reproducibility and confounder assessment.

## 5. Conclusions

This study provides preliminary evidence that standardized LAB-derived tongue and facial image features, particularly when combined with demographic information, may capture group-level differences across clinician-assigned clinical reference groups. These findings suggest that automated tongue and facial phenotyping may serve as a complementary and exploratory source of information in psychiatric research, rather than a stand-alone diagnostic tool. Because demographic variables contributed substantially to classification performance, the observed predictive patterns should be interpreted as exploratory group-level associations rather than diagnostic biomarkers. Future studies using balanced samples, structured diagnostic interviews, medication information, external validation cohorts, and prospective designs are needed before these features can be considered for clinical decision support.

## Figures and Tables

**Table 1:** Demographic characteristics of the study sample.

Characteristic	HC (n = 84)	Depression (n = 246)	Schizophrenia (n = 419)	Total (n = 749)
Age, mean ± SD (years)	42.96 ± 13.30	28.94 ± 18.59	53.92 ± 11.82	44.49 ± 18.45
Height, mean ± SD (cm)	164.39 ± 8.04	165.23 ± 8.34	166.30 ± 8.30	165.73 ± 8.30
Weight, mean ± SD (kg)	64.39 ± 16.45	60.37 ± 13.04	70.72 ± 13.58	66.61 ± 14.54
Male, n (%)	57 (67.9%)	176 (71.5%)	197 (47.0%)	430 (57.4%)
Female, n (%)	27 (32.1%)	70 (28.5%)	222 (53.0%)	319 (42.6%)

Note: Values are presented as mean ± standard deviation for continuous variables and number (%) for categorical variables. Diagnostic group assignment was based on clinician-assigned routine clinical diagnoses, which were used as clinical reference labels in the present study.

**Table 2:** Multiclass classification performance across feature sets over five repeated stratified splits.

Feature Set	Accuracy	Bal. Accuracy	Macro F1	AUC	AUPRC	ΔAUC vs. Demo
Demographics only	0.754 ± 0.036	0.631 ± 0.046	0.639 ± 0.046	0.855 ± 0.024	0.805 ± 0.028	—
LAB only	0.704 ± 0.040	0.554 ± 0.052	0.552 ± 0.051	0.859 ± 0.035	0.761 ± 0.053	+0.004
TCM-only	0.538 ± 0.043	0.467 ± 0.055	0.456 ± 0.049	0.682 ± 0.043	0.604 ± 0.037	−0.173
LAB + Demographics	0.782 ± 0.035	0.708 ± 0.042	0.697 ± 0.038	0.912 ± 0.027	0.858 ± 0.016	+0.057
LAB + TCM	0.703 ± 0.073	0.588 ± 0.063	0.588 ± 0.067	0.850 ± 0.052	0.760 ± 0.060	−0.005
LAB + Demographics + TCM	0.784 ± 0.051	0.705 ± 0.044	0.699 ± 0.045	0.912 ± 0.032	0.858 ± 0.027	+0.057

Note: Values are presented as mean ± standard deviation across five random seeds. Balanced accuracy represents the average recall across classes. Macro-F1 denotes the unweighted mean F1 score across classes. AUC was computed using a weighted-average scheme. ΔAUC vs. Demo indicates the difference in AUC relative to the demographic-only model.

**Table 3:** Pairwise binary classification performance.

Binary Task	Accuracy	Bal. Accuracy	F1 Score	AUC	AUPRC
HC vs. Depression	0.752 ± 0.059	0.743 ± 0.030	0.815 ± 0.059	0.817 ± 0.054	0.925 ± 0.033
HC vs. Schizophrenia	0.892 ± 0.046	0.898 ± 0.023	0.931 ± 0.032	0.952 ± 0.030	0.990 ± 0.006
Depression vs. Schizophrenia	0.874 ± 0.026	0.870 ± 0.026	0.898 ± 0.022	0.926 ± 0.019	0.935 ± 0.034

Note: Values are presented as mean ± standard deviation across five random seeds. Pairwise models were trained independently for each diagnostic contrast using the same repeated stratified splitting procedure. AUC denotes the area under the receiver operating characteristic curve, and AUPRC denotes the area under the precision–recall curve.

## Data Availability

The raw data supporting the conclusions of this article will be made available from the authors by reasonable request.

## Supplementary Materials

The following supporting information can be downloaded at: https://www.mdpi.com/article/10.3390/bs16081442/s1, Table S1: Complete list of variables included in each feature set used for model development; Table S2: Mean normalized confusion matrix for the multiclass LAB + Demographics + TCM model across five seeds; Table S3: Class-specific performance of the multiclass LAB + Demographics + TCM model across five repeated stratified splits; Table S4: Top-15 variables from permutation importance analysis of the multiclass LAB + Demographics + TCM model.

## Abbreviations

The following abbreviations are used in this manuscript:

AutoML	Automated Machine Learning
HC	Healthy Control
TCM	Traditional Chinese Medicine
