# Supplementary material for: From Traditional Inspection to Quantitative Imaging: Tongue and Facial Color Features for Automated Machine Learning-Driven Depression and Schizophrenia Classification
Source: Behav Sci (Basel). 2026 Aug 20;16(8):1442. doi: 10.3390/bs16081442 (PMC13510007; doi:10.3390/bs16081442)
Supplement: Supplementary file 1 [file behavsci-16-01442-s001.zip › behavsci-4305814-supplementary.pdf]

Supplementary Table S1

Complete list of variables included in each feature set used for model development.

No.	Category	Variable Name	Variable Type
1	Demographics	age	Continuous
2	Demographics	height	Continuous
3	Demographics	weight	Continuous
4	Demographics	sex_male	Binary
5	Demographics	sex_female	Binary
6	LAB	complexion_result_l	Continuous
7	LAB	complexion_result_a	Continuous
8	LAB	complexion_result_b	Continuous
9	LAB	gloss_result_l	Continuous
10	LAB	gloss_result_a	Continuous
11	LAB	gloss_result_b	Continuous
12	LAB	lip_color_l	Continuous
13	LAB	lip_color_a	Continuous
14	LAB	lip_color_b	Continuous
15	LAB	tongue_color_l	Continuous
16	LAB	tongue_color_a	Continuous
17	LAB	tongue_color_b	Continuous
18	LAB	coating_color_l	Continuous
19	LAB	coating_color_a	Continuous
20	LAB	coating_color_b	Continuous
21	TCM	tongue_size_swollen_tongue	Binary
22	TCM	tongue_size_thin_tongue	Binary
23	TCM	tongue_coating_thick_coating	Binary
24	TCM	tongue_coating_thin_coating	Binary
25	TCM	tongue_fissure_fissure	Binary
26	TCM	tongue_fissure_absent	Binary
27	TCM	tongue_teeth_mark_teeth_mark	Binary
28	TCM	tongue_teeth_mark_absent	Binary
29	TCM	tongue_greasy_coating_greasy_coating	Binary
30	TCM	tongue_greasy_coating_absent	Binary
31	TCM	complexion_result_normal	Binary
32	TCM	complexion_result_pale_complexion	Binary
33	TCM	complexion_result_red_complexion	Binary
34	TCM	complexion_result_yellow_complexion	Binary
35	TCM	gloss_result_glossy	Binary
36	TCM	gloss_result_dull	Binary
37	TCM	lip_color_red	Binary
38	TCM	lip_color_dark_red	Binary
39	TCM	tongue_color_red_tongue	Binary

40	TCM	tongue_color_deep_red_tongue	Binary
41	TCM	tongue_color_pale_white_tongue	Binary
42	TCM	tongue_color_dark_red_tongue	Binary
43	TCM	tongue_color_pale_red_tongue	Binary
44	TCM	coating_color_yellow_coating	Binary
45	TCM	coating_color_white_coating	Binary

Supplementary Table S2

Mean normalized confusion matrix for the multiclass LAB + Demographics + TCM model across five seeds.

True class	Predicted HC	Predicted Depression	Predicted Schizophrenia
HC	0.538 ± 0.054	0.231 ± 0.077	0.231 ± 0.054
Depression	0.141 ± 0.065	0.681 ± 0.075	0.178 ± 0.062
Schizophrenia	0.089 ± 0.087	0.016 ± 0.011	0.895 ± 0.088

Note: Values are presented as mean ± standard deviation across five random seeds. Rows represent true classes and columns represent predicted classes. Values are row-normalized proportions; therefore, each row sums to approximately 1.00. HC, healthy control.

Supplementary Table S3

Class-specific performance of the multiclass LAB + Demographics + TCM model across five repeated stratified splits.

Class	Precision	Recall	F1 score
HC	0.420 ± 0.098	0.538 ± 0.054	0.464 ± 0.070
Depression	0.862 ± 0.044	0.681 ± 0.075	0.760 ± 0.059
Schizophrenia	0.854 ± 0.037	0.895 ± 0.088	0.873 ± 0.055

Note: Values are presented as mean ± standard deviation across five random seeds. Precision, recall, and F1 score were calculated separately for each class using the held-out test set for each repeated split. HC, healthy control.

Supplementary Table S4

Top 15 variables from permutation importance analysis of the multiclass LAB + Demographics + TCM model.

Rank	Feature	Importance, Mean ± SD	N_Seeds
1	age	0.2012 ± 0.0524	5
2	coating_color_l	0.1021 ± 0.0363	5
3	gloss_result_a	0.0294 ± 0.0330	3
4	lip_color_l	0.0093 ± 0.0075	3
5	gloss_result_l	0.0067 ± 0.0050	3
6	weight	0.0063 ± 0.0040	3
7	tongue_fissure_fissure	0.0050 ± 0.0053	3
8	complexion_result_pale_complexion	0.0048 ± 0.0057	4
9	tongue_size_swollen_tongue	0.0043 ± 0.0046	4
10	tongue_coating_thick_coating	0.0033 ± 0.0019	3
11	tongue_size_thin_tongue	0.0031 ± 0.0030	3

12	complexion_result_a	0.0027 ± 0.0047	3
13	lip_color_dark_red	0.0024 ± 0.0042	3
14	coating_color_yellow_coating	0.0019 ± 0.0019	3
15	tongue_color_pale_red_tongue	0.0008 ± 0.0023	4

Note: Variables are ranked by mean permutation importance across repeated runs. Importance values are presented as mean ± standard deviation. N seeds indicates the number of random-seed runs in which the variable was retained among the top-ranked features. Feature names are reported as used in the modeling pipeline.
